# Evolution of a Pathogen: A Comparative Genomics Analysis Identifies a Genetic Pathway to Pathogenesis in *Acinetobacter*

**DOI:** 10.1371/journal.pone.0054287

**Published:** 2013-01-24

**Authors:** Jason W. Sahl, John D. Gillece, James M. Schupp, Victor G. Waddell, Elizabeth M. Driebe, David M. Engelthaler, Paul Keim

**Affiliations:** 1 Department of Pathogen Genomics, Translational Genomics Research Institute, Flagstaff, Arizona, United States of America; 2 Arizona Department of Health Services, Bureau of State Laboratory Services, Phoenix, Arizona, United States of America; 3 Center for Microbial Genetics and Genomics, Northern Arizona University, Flagstaff, Arizona, United States of America; University of Edinburgh, United Kingdom

## Abstract

*Acinetobacter baumannii* is an emergent and global nosocomial pathogen. In addition to *A. baumannii*, other *Acinetobacter* species, especially those in the *Acinetobacter calcoaceticus*-*baumannii* (*Acb*) complex, have also been associated with serious human infection. Although mechanisms of attachment, persistence on abiotic surfaces, and pathogenesis in *A. baumannii* have been identified, the genetic mechanisms that explain the emergence of *A. baumannii* as the most widespread and virulent *Acinetobacter* species are not fully understood. Recent whole genome sequencing has provided insight into the phylogenetic structure of the genus *Acinetobacter*. However, a global comparison of genomic features between *Acinetobacter* spp. has not been described in the literature. In this study, 136 *Acinetobacter* genomes, including 67 sequenced in this study, were compared to identify the acquisition and loss of genes in the expansion of the *Acinetobacter* genus. A whole genome phylogeny confirmed that *A. baumannii* is a monophyletic clade and that the larger *Acb* complex is also a well-supported monophyletic group. The whole genome phylogeny provided the framework for a global genomic comparison based on a blast score ratio (BSR) analysis. The BSR analysis demonstrated that specific genes have been both lost and acquired in the evolution of *A. baumannii*. In addition, several genes associated with *A. baumannii* pathogenesis were found to be more conserved in the *Acb* complex, and especially in *A. baumannii*, than in other *Acinetobacter* genomes; until recently, a global analysis of the distribution and conservation of virulence factors across the genus was not possible. The results demonstrate that the acquisition of specific virulence factors has likely contributed to the widespread persistence and virulence of *A. baumannii*. The identification of novel features associated with transcriptional regulation and acquired by clades in the *Acb* complex presents targets for better understanding the evolution of pathogenesis and virulence in the expansion of the genus.

## Introduction

*Acinetobacter baumannii* is a nosocomial pathogen implicated with septicemia, pneumonia, and death [1], [2], [3]. *A. baumannii* is truly a global pathogen, as it has been isolated from hospitals throughout the world [4], [5], [6], [7], as well as in wounded soldiers serving in Iraq [8], [9], [10] and Afghanistan [11]. *Acinetobacter* is a genus in the phylum Proteobacteria, family *Moraxellaceae*, consisting of 26 named species (http://www.bacterio.cict.fr/a/acinetobacter.html). *Acinetobacter* spp. are found in most soil and water samples [12], but are also a major source of nosocomial infections [13]; the natural environments for the pathogens *A. baumannii* and *A. nosocomialis* are not currently known [14]. Although most nosocomial *Acinetobacter* infections are associated with *A. baumannii* [15], other species have also been associated with human disease. For example, *A. ursingii* has also been associated with nosocomial bloodstream infections [16], [17].

Our understanding of the pathogenesis of *A. baumannii* is largely based on the ad-hoc analyses of putative virulence factors. Much of the pathogenesis research in *A. baumannii* has focused on biofilm formation [18], [19], although no conclusive link between biofilm formation and infection has been established [20]. Recent studies have demonstrated that iron acquisition systems in *A. baumannii* are important virulence factors [21]. Three distinct siderophore systems associated with iron acquisition have been described in *A. baumannii* [22], [23]; the most well characterized of these systems is acinetobactin, which shares homology to a plasmid-encoded siderophore in *Vibrio anguillarum* [24], [25]. A recent study demonstrated that the proteins BasD and BauA, which are necessary for acinetobactin synthesis, are required for the pathogen to persist and ultimately kill host epithelial cells [26].

One of the primary concerns of *A. baumannii* as a nosocomial pathogen is its resistance to antimicrobials [7]. Large antibiotic resistance islands (RI), including the 86-kb RI in strain AYE [27], have been characterized in *A. baumannii*. Broad-spectrum beta-lactamase genes [28], which are part of the carbapenem-hydrolysing class D beta-lactamases (CHDLs) subgroup [29], have been identified in *A. baumannii*, including bla_OXA-51_ [30], bla_OXA-23_ [31], and the plasmid-encoded gene bla_OXA-58_ [32]. In addition, the expression of efflux pump systems, such as AdeFGH [33], AdeABC [34], AdeIJK [35], and AdeM [36] has been associated with multidrug resistance in *A. baumannii*.

Much of the research on the genus *Acinetobacter* has focused on genomes in the *Acinetobacter calcoaceticus-baumannii* (*Acb*) complex [37]. This complex includes *A. baumannii*, *A. nosocomialis* (originally genomic species (gen. sp.) 13TU [38]), *A. pittii* (originally gen. sp. 3 [38]), *A.* sp. DR1 [39], and *A. calcoaceticus*. *A. calcoaceticus* and *A.* sp. DR1 have not been implicated in serious human infection [14]. A recent study of *Acinetobacter* bloodstream infections in the United States demonstrated that the most common nosocomial infections were caused by *A. baumannii* (63% of cases), *A. nosocomialis* (21% of cases), and *A. pittii* (8% of cases) [40].

From a genomics perspective, most analyses of whole genome sequence data have focused on multi-drug resistant (MDR) isolates [41], [42], [43], [44], [45], although additional studies have considered evolutionary relationships of *A. baumannii* [46], [47]. In this study, a large-scale whole genome sequence analysis was performed on a set of previously characterized (n = 69), as well as newly sequenced (n = 67), *Acinetobacter* genomes. These data were used to identify the genomic diversity of the genus *Acinetobacter* and understand the flow of genetic information between species in the genus. Prior to whole genome sequence analysis, studying the conservation of diverse genes across the genus was not possible. This information is not only pertinent from a pathogenesis perspective, but may also aid in the identification of targets important for diagnostic, therapeutic and vaccine development.

## Methods and Materials

### Strain selection/clinical data

Sixty-seven *Acinetobacter* genomes were sequenced from a variety of human and environmental sources (Table S1). Isolates were selected to capture a broad range of genetic, geographic, and temporal diversity and were not chosen based on specific clinical outcomes. Species designations were applied by the position of the isolate in the phylogeny and were not based on clinical typing methods.

### DNA isolation, sequencing, assembly

*Acinetobacter* isolates were grown on nutrient agar for 24 hours. Genomic DNA was extracted following the manufacturer's protocol for Gram-negative bacteria in the Qiagen DNeasy Blood and Tissue kit (Cat # 69504). DNA samples were prepared for multiplexed, paired end sequencing following the manufacturer's protocol. For each isolate, 1–5 ug of dsDNA in 200 ul was sheared and then purified using the QIAquick PCR Purification kit (Cat #28106, Qiagen,Valencia, CA). Enzymatic processing of the DNA followed the guidelines as described in the Illumina protocol, but enzymes for processing were obtained from New England Biolabs (Cat #E6000L, New England Biolabs, Ipswich, MA) and the oligonucleotides and adaptors were obtained from Illumina (Cat #PE-400-1001). After ligation of the adaptors, the DNA was run on a 2% agarose gel for 2 hours, after which a gel slice containing 500–600 bp fragments of each DNA sample was isolated and purified using the QIAquick Gel Extraction kit (Cat #28706, Qiagen, Valencia, CA). Individual libraries were quantified with qPCR on the ABI 7900HT (Part #4329001, Life Technologies Corporation, Carlsbad, CA) using the Kapa Library Quantification Kit (part # KK4832 or KK4835, Kapa Biosystems, Woburn, MA). Based on the individual library concentrations, equimolar pools of libraries were prepared at a concentration of at least 1 nM. The pooled libraries were sequenced on the Illumina GA-IIx using the “Genomic DNA sequencing primer V2” protocol for 36 cycles. A 100 bp paired-end run was used for all isolates.

Paired-end sequence reads were assembled with Velvet [48], in conjunction with the VelvetOptimiser (http://bioinformatics.net.au/software.velvetoptimiser.shtml). Contigs shorter than 200 nucleotides were filtered from the assembly. Errors in each assembly were corrected with iCORN [49]. Assembly statistics are detailed in Table S1.

### Whole genome phylogeny of the genus *Acinetobacter*

136 genomes were processed with the kSNP analysis tool [50], which generated a multiple sequence alignment based on single nucleotide polymorphisms (SNPs). This alignment included SNPs from 69 reference genomes (Table S2) and 67 genomes sequenced in this study (Table S1). Singleton and homoplastic SNPs were removed from the alignment with noisy [51] using the “nogap” setting. A tree was inferred on this reduced alignment with FastTree2 [52], using the following parameters: -spr 4 -mlacc 2 –slownni. The tree was rooted by *A. radioresistens* in FigTree (http://beast.bio.ed.ac.uk/FigTree).

### Blast score ratio (BSR) analysis

Contigs from draft assemblies were concatenated with a linker (NNNNNCACACACTTAATTAATTAAGTGTGTGNNNNN) inserted between each contig; this linker contains a start/stop codon in all 6 frames. Coding regions (CDSs) were predicted using Glimmer3 [53]. CDSs from each genome were concatenated and then de-replicated by clustering with USEARCH [54], using an ID of 0.8 and an IDDEF value of 3. Low-density clusters (n<4) were removed to reduce the size of the dataset. Each representative cluster was then translated with transeq [55]; the translated sequence was then aligned against each genome in the dataset with TBLASTN [56]. The query bit score for each genome alignment was divided by the maximum bit score in all genomes to obtain the blast score ratio (BSR) [57]; the BSR value can range from 1.0 (100% ID across 100% of the peptide) to 0 (no significant alignment).

### *In silico* gene screen

To identify the conservation of specific genes associated with virulence (Table S3) across the genus *Acinetobacter*, the peptide sequence for each virulence factor was aligned against all sequenced genomes with TBLASTN. The BSR values across groups were visualized with the multi-experiment viewer [58]. Raw BSR values for each marker screened in this study are listed in Table S4.

### Multi-locus sequence typing (MLST) phylogeny

For a comparison to the whole-genome phylogeny, a tree was inferred from concatenated MLST sequences. Sequences from MLST markers were downloaded (*gltA*, *gyrB*, *gdhB*, *recA*, cpn60, *gpi*, *rpoD*) from the *A. baumannii* pubMLST database (pubmlst.org/abaumannii). Sequences were extracted from BLAST alignments, concatenated, aligned with MUSCLE [59], and a phylogeny was inferred with FastTree2, as has been done previously [60].

### 16S rRNA gene sequence analysis

For an additional comparison to the whole genome phylogeny, a tree was inferred from an alignment of 16S rRNA gene sequences. Sequences were extracted from all genomes with SSU-ALIGN (http://selab.janelia.org/software.html), which is based on a covariance model implemented in Infernal [61]. These sequences were combined with reference sequences in the Greengenes database [62]. Gene sequences were exported from ARB [63] and all sequences were aligned with SSU-ALIGN. Homoplastic and singleton SNPs were removed from the alignment with noisy. A tree was inferred with FastTree2, using the same parameters as with the whole genome phylogeny. Monophyletic clades were collapsed in ARB. Sequences from a basal branch in the Gammaproteobacteria were used to root the phylogeny.

## Results

### Whole genome sequencing of 67 new *Acinetobacter* genomes

In order to expand the *Acinetobacter* phylogeny, 67 genomes were sequenced and analyzed. While most sequenced isolates (n = 47) were identified as *A. baumannii*, genomes (n = 20) from other species were included. These genomes represent a significant addition of genomic data from the genus and will serve as a valuable resource for future genomic studies.

### Whole genome phylogeny of the genus *Acinetobacter*

**Figure 1:**
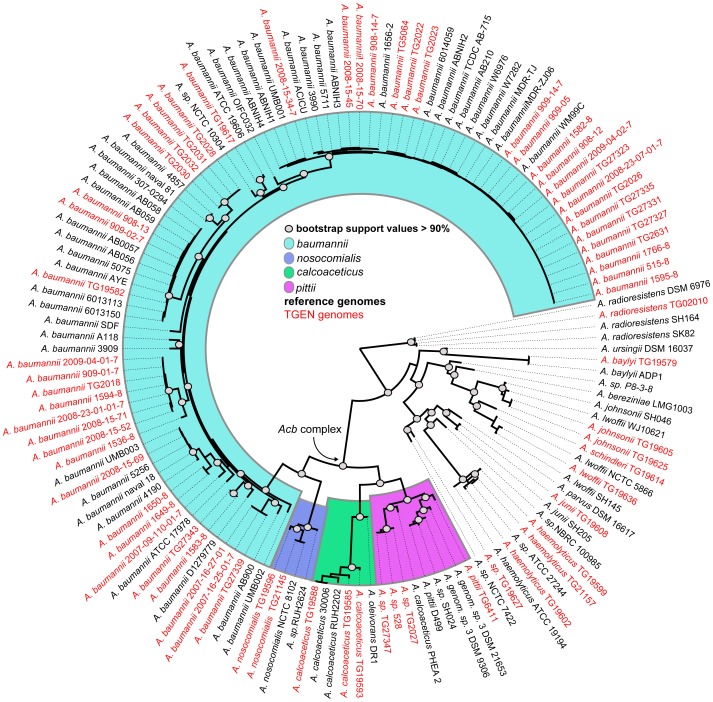
A whole genome phylogeny of 136 sequenced genomes in the genus *Acinetobacter*. The phylogeny was inferred with FastTree2 [52] on a single nucleotide polymorphism (SNP) matrix alignment calculated with kSNP [50] and filtered with noisy [51]. The phylogeny was rooted with *A. radioresistens*. Genomes sequenced in the current study are shown in red. Genomes in the *Acinetobacter calcoaceticus-baumannii* (*Acb*) complex are colored by clade.

A whole genome phylogeny was inferred on ∼200,000 single nucleotide polymorphisms (SNPs) identified by kSNP (Figure 1). This phylogeny represents the most comprehensive global phylogeny of *Acinetobacter* based on whole genome sequencing and is consistent with a recent study of *Acinetobacter* evolution [64]. The results confirm that *A. baumannii* genomes comprise a monophyletic clade that is part of the larger *Acinetobacter calcoaceticus-baumannii* (*Acb*) complex. In addition to pathogens, the *Acb* complex contains environmental isolates not associated with serious human disease [65]; the three isolates sequenced in this study (*A. calcoaceticus* TG19593, TG19585, TG19588) from the environmental clade, including *A. calcoaceticus* and *A.* sp. DR1, are soil isolates not associated with serious human infection.

The phylogeny was rooted with *A. radioresistens*, which has also shown to be the root for a tree inferred from *rpoB* sequences [38] and from an amplified fragment length polymorphism (AFLP) analysis [66]. The 16S rRNA gene sequence phylogeny inferred in this study supports *A. radioresistens* as the most basal clade of previously sequenced *Acinetobacter* spp. (Figure S1). A phylogeny inferred from a concatenation of multi-locus sequence typing (MLST) markers revealed a similar topology to the whole genome phylogeny in the *Acb* complex (Figure S2).

### Genome size differences in *Acinetobacter* species

To determine differences in genome size at specific nodes in the phylogeny, genome assembly sizes (Table S1) were compared between the *Acinetobacter* root (*A. radioresistens*), the *Acb* complex, and the remaining *Acinetobacter* clade (Figure 1). The *radioresistens* clade had the smallest average genome size (3.21, 95% CI+/−0.13 mb), while the *Acb* complex had the largest (3.94, 95% CI+/−0.05 mb); the remaining *Acinetobacter* clade was intermediate in size (3.5, 95% CI+/−0.13 mb).

### Gene gain/loss in the *Acb* complex

**Figure 2:**
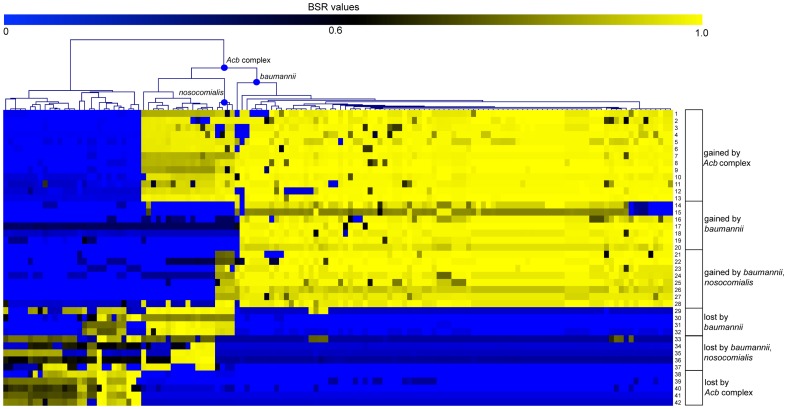
A heatmap of blast score ratio (BSR) [**57**] values for branch specific regions in the *Acb* complex. BSR values were visualized with the multi-experiment viewer [58]. Samples were clustered using an average linkage clustering algorithm. Numbers for each feature correlate with features described in Table 1. Raw data values are shown in Table S5.

Based on the average genome size, the *Acb* complex has acquired a complement of genes compared to all other sequenced *Acinetobacter* species. To identify genes that were gained and lost in the *Acb* complex, a blast score ratio (BSR) analysis with peptide sequences was performed. For a gene to be considered as acquired by a lineage, we required a BSR value ≥0.8 in >85% of targeted genomes and a BSR value <0.4 in >95% of non-targeted genomes; a BSR value = 0.8 is approximate to 80% peptide identity over 100% of the peptide length [67]. Genomic targets that met these criteria were tabulated (Table 1). The peptide sequence for each gene was aligned against all genomes (n = 136) with TBLASTN [56]. The results demonstrate that each gene is specific to each targeted group based on the BSR analysis (Figure 2).

**Table 1:** Annotation details of lost and acquired genes in the evolution of *A. baumannii*.

feature	locus_tag	annotation	clade*	type
1	ABAYE2283	lipase	*Acb*	gain
2	ABAYE0080	signal peptide	*Acb*	gain
3	ABAYE3752	porin	*Acb*	gain
4	ABAYE3753	efflux pump membrane transporter	*Acb*	gain
5	ABAYE3761	autoinducer synthesis protein	*Acb*	gain
6	ABAYE2829	aldose 1-epimerase	*Acb*	gain
7	ABAYE1524	Lactonase	*Acb*	gain
8	ABAYE1477	chorismate mutase	*Acb*	gain
9	ABAYE2143	hypothethical	*Acb*	gain
10	ABAYE0743	hypothethical	*Acb*	gain
11	ABAYE1358	multidrug resistance protein	*Acb*	gain
12	ABAYE1778	multidrug resistance transporter	*Acb*	gain
13	ABAYE0223	AraC family transcriptional regulator	*Acb*	gain
14	ACICU_01049	hypothethical	b	gain
15	ABAYE2708	hypothethical	b	gain
16	HMPREF0022_02462	hypothethical	b	gain
17	ACICU_02424	ATPase	b	gain
18	ABAYE1316	GntR family transcriptional regulator	b	gain
19	ABAYE2003	hypothethical	b	gain
20	AbauAB05_010100017737	hypothethical	b	gain
21	AbauAB059_010100020508	hypothethical	b,n	gain
22	HMPREF0022_00070	hypothethical	b,n	gain
23	ABZJ_00026	hypothethical	b,n	gain
24	AbauAB0_010100001652	hypothethical	b,n	gain
25	ABAYE2456	beta-lactamase	b,n	gain
26	HMPREF0021_00524	hypothethical	b,n	gain
27	ABAYE0983	hypothethical	b,n	gain
28	ABAYE1931	GntR family transcriptional regulator	b,n	gain
29	HMPREF0012_03541	hypothethical	b	loss
30	HMPREF0012_00124	glyoxylase	b	loss
31	HMPREF0012_00560	hypothethical	b	loss
32	HMPREF0012_00562	RNA polymerase sigma factor	b	loss
33	HMPREF0023_2071	bile acid:sodium symporter	b,n	loss
34	HMPREF0013_03409	hypothethical	b,n	loss
35	HMPREF0013_03179	hypothethical	b,n	loss
36	HMPREF0013_03184	3-hydroxyisobutyrate dehydrogenase	b,n	loss
37	HMPREF0012_01063	hypothethical	b,n	loss
38	HMPREF0026_02170	urea ABC transporter	*Acb*	loss
39	HMPREF0026_00688	tellurite resistance protein	*Acb*	loss
40	HMP0015_2881	hypothethical	*Acb*	loss
41	HMPREF0026_01273	methanol dehydrogenase regulatory protein	*Acb*	loss
42	HMPREF0023_2956	amidase	*Acb*	loss

* *Acb* = *Acinetobacter calcoaceticus-baumannii*, b = *baumannii*, n = *nosocomialis*.

Genes unique to the *Acb* complex (Table 1, Figure 2) include a multidrug resistance protein, a multidrug resistance transporter, and an AraC-family transcriptional regulator. Genes either not acquired or lost by the complex include a tellurite-resistance gene and a urea ABC transporter (Table 1) that shares homology (>80% identity over 100% of the peptide length) to membrane transporters in other Pseudomonads. Additional genes shared homology to hypothetical proteins with no known function.

Genes were also identified that are conserved in the *nosocomialis*-*baumannii* clade, but are absent from other genomes in the *Acb* complex. Genes unique to this clade include a class A beta-lactamase (TEM-1), a GntR-family transcriptional regulator (ABAYE1931), and conserved hypothetical proteins (Table 1); the acquired transcriptional regulator shares homology (56% ID over 100% of the peptide length) with a transcriptional regulator (bgla_2g16570) in the plant pathogen, *Burkholderia gladioli* [68]. Genes lost by this sub-clade include a bile acid sodium symporter associated with resistance to arsenic compounds [69] (Table 1).

Genes unique to the *A. baumannii* clade include hypothetical proteins and a GntR family (FCD domain) transcriptional regulator (ABAYE1316); this regulator shares homology (39% ID over 96% of the peptide length) with a regulator (PST_2058) in the opportunistic pathogen, *Pseudomonas stutzeri* [70]. Genes lost in *A. baumannii* include a glyoxylase/bleomycin resistance gene and a specific ECF-type RNA polymerase sigma factor. The functions of acquired hypothetical proteins in the *Acb* complex are not currently known; however, several of these peptides share homology with genes in known pathogens (Table S5).

### Siderophore distribution and conservation in the genus *Acinetobacter*

**Figure 3:**
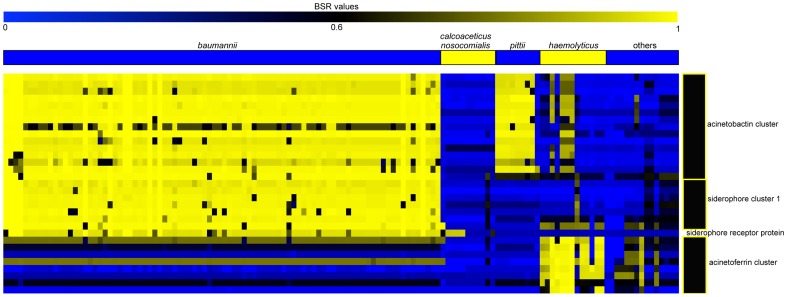
A heatmap of blast score ratio (BSR) [**57**] values for iron acquisition genes in *Acinetobacter*. BSR values were visualized with the multi-experiment viewer [58]. Accession details for each gene in specific iron acquisition systems are shown in Table S3, with raw data shown in Table S5.

One identified pathogenic mechanism of *A. baumannii* infection is through the expression of siderophores [24]. However, the conservation of siderophore genes across the *Acinetobacter* genus is not currently known. The acinetobactin iron acquisition cluster, which consists of 25 kb of sequence in seven operons [71], in *A. baumannii* AYE (Table S3) [27], was aligned against all *Acinetobacter* genomes analyzed in this study with TBLASTN. Genes screened included BasD [25], and BasE, which activates the 2,3-dihydroxybenzoic acid molecule and transfers it to BasF [72]. The acinetobactin cluster was highly conserved in *A. baumannii* and the clade that includes *A. pittii* (Figure 3); homologous peptides were also observed in several *A. haemolyticus* isolates, as has been previously observed [72]. The *A. baumannii* clade also contains siderophore cluster 1 (ABAYE2001-2008) [21] that appears to be unique to *A. baumannii* (Figure 3). Components of the acinetoferrin system are conserved in the *A. haemolyticus* clade [73]; four components of the acinetoferrin cluster are homologous to peptides in *A. baumannii* (Figure 3). In addition to siderophore genes, the *A. baumannii-nosocomialis* clade also contains a unique TonB-dependent siderophore receptor protein (ABAYE1644) (Figure 3); these proteins are involved in sensing extra-cellular signals and responding through transcriptional regulation [74].

### Carbapenem-hydrolyzing oxacillinases (OXA)

**Figure 4:**
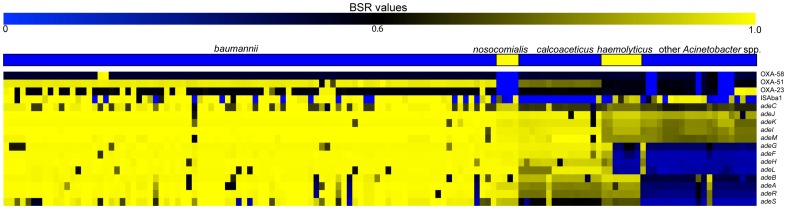
A heatmap of blast score ratio (BSR) [**57**] values for efflux pump and beta-lactamase genes identified in *Acinetobacter*. BSR values were visualized with the multi-experiment viewer [58]. Accession details for each gene are shown in Table S3, with raw data shown in Table S5.

Antibiotic resistance is common in nosocomial *A. baumannii* infections [75]. One mechanism of resistance to beta-lactams, including carbapenems, in *A. baumannii* is through the expression of OXA-type enzymes [14]. To determine their distribution, OXA genes (Table S3) were informatically screened against all sequenced *Acinetobacter* genomes. Only bla_OXA-51-like_ [30] genes were found to be conserved across the majority of *Acinetobacter* spp. (Figure 4); genomes from the *nosocomialis* and *baylyi* clades lack a bla_OXA-51_ homolog (Table S4). The bla_OXA-51-like_ genes are specific to each species and have been proposed as a method of positively identifying *A. baumannii* isolates [76]. An *in silico* screen demonstrated that bla_OXA-51-like_ primers [76] not only align with all *A. baumannii* genomes (n = 89) analyzed in this study, but do not significantly align with any other *Acinetobacter* spp. (n = 47), including non-*baumannii* genomes in the *Acb* complex.

In *A. baumannii* isolates that only contain bla_OXA-51-like_ genes, the presence of the insertion element IS*Aba*1 is required for carbapenem resistance [77]. For example, the multi-drug susceptible genomes AB058, A118, AB900 and AB307-0294 are all bla_OXA-51-like_ positive and IS*Aba*1 negative (Table S4). While IS*Aba*1 appears to be conserved in *A. baumannii*, a BSR analysis demonstrated that the insertion element is also present in other *Acinetobacter* spp. (Figure 4).

## Discussion

The genus *Acinetobacter* contains a mixture of non-pathogenic environmental isolates and wide-spread nosocomial pathogens [78]. Although other *Acinetobacter* spp. have been implicated in human infection, *A. baumannii* appears to be the most widespread and virulent. With the advent of whole-genome sequencing and large-scale comparative analyses, the genomic differences between *A. baumannii* and related isolates can now be identified. The current study represents the most comprehensive comparative analysis of the conservation and distribution of virulence factors across the *Acinetobacter* genus performed to date.

The *Acinetobacter* whole genome phylogeny confirms that the *A. calcoaceticus*-*baumannii* (*Acb*) complex is monophyletic (Figure 1). Isolates from this complex are frequently associated with nosocomial infections [40], [79]. However, isolates from the *calcoaceticus* clade (Figure 1) are typically environmental isolates not associated with serious human infection [14]. In addition, *A. pittii* strain PHEA-2, was isolated from industry wastewater [80]. From a clinical perspective, the identification of *Acb* complex isolates, which include both environmental isolates and human pathogens, may be misleading from a treatment perspective [14]. Genomic regions identified in this study and guided by a whole genome phylogeny may provide better discrimination between related stains of varying clinical significance. The identification of clinical isolates based on acquired genes on specific phylogenetic branches may help to develop diagnostics that can accurately classify infections from nosocomial *Acinetobacter* pathogens.

Whole genome sequence analysis of 136 *Acinetobacter* genomes has provided a comprehensive view of *Acinetobacter* evolution. Based on the analysis of several conserved genes, *A. radioresistens* is the most basal lineage of sequenced *Acinetobacter* genomes. In addition, the *A. radioresistens* clade was shown to have the smallest average genome size where the *Acb* complex has the largest. Although genome reduction is generally associated with a pathogenic lifestyle [81], genome expansion in pathogens has also been observed [82]. While the functional roles of all acquired genes in *A. baumannii* have not yet been determined, they may be associated with the persistence and virulence of *A. baumannii* in hospital environments.

*A. baumannii* thrives in hospital settings, largely due to its persistence on abiotic surfaces [83]. One mechanism for *A. baumannii* persistence in hospital settings is the presence of a putative tip adhesion gene, *csuE*. The *csuE* gene (ABK1_1276) is involved in pilus and biofilm formation [19] and is largely conserved (BSR>0.8) in the *A. baumannii* (>92% presence), *A. nosocomialis* (100% presence), and *A. pittii* (67% presence) clades; *csuE* is absent (BSR<0.4) from environmental isolates in the *calcoaceticus* clade as well as *Acinetobacter* genomes not in the *Acb* complex (Table S5). The presence of this gene has been associated with the persistence of *A. baumannii* on abiotic surfaces, such as plastic and glass [19]. The lack of a homologous gene in non-*Acb* isolates could explain why pathogens in the *Acb* complex persist in the hospital environment. Furthermore, *A. baumannii* can survive desiccation much better than most other *Acinetobacter* spp. [84]; however, *A. radioresistens* has been shown to be perhaps the most desiccation-tolerant *Acinetobacter* spp. [85]. This suggests a mechanism for attachment, as well as persistence, on abiotic hospital surfaces.

The role of siderophores in bacterial pathogenesis is well characterized [86]. When *A. baumannii* invades the host, one mechanism of persistence and toxicity is the acinetobactin iron-acquisition system [24]. Although not unique to *A. baumannii*, the acinetobactin cluster is well conserved in the species and is likely a contributing factor in *A. baumannii* survival and pathogenesis. Three unique iron-acquisition systems have been identified in *A. baumannii* including siderophore cluster 1, which appears to be unique to *A. baumannii* (Figure 3). In a study of gene expression in iron-limited media, all three iron acquisition systems identified in *A. baumannii* were up regulated [21]. The presence of multiple iron acquisition systems could provide a competitive advantage for the pathogen over other host microbiota.

Antibiotic resistance may be the most troubling aspect in the evolution of the *Acb* complex. A unique gene acquired in the evolution of *A. baumannii*, compared to other *Acinetobacter* spp., is a narrow-spectrum TEM-1 beta-lactamase, which is highly conserved in *A. baumannii* and *A. nosocomialis*; however, the clinical significance of this gene acquisition is unclear [14]. What is of great concern is the resistance of *A. baumannii* to broad-spectrum antibiotics, such as fluoroquinolones, especially in outbreak events [87]. Beta-lactamase genes are conserved in *A. baumannii* [88], which allow the bacterium to persist in the host and resist treatment therapies. Efflux pumps are also important mechanisms for antibiotic resistance in *A. baumannii* [42]; a comparative analysis demonstrated that *A. baumannii* contains an assortment of efflux pump genes that are not well conserved outside of the *Acb* complex (Figure 4). Although additional mechanisms of resistance, including 16S rRNA methylation [89], have been described for *A. baumannii*, they are more difficult to identify through comparative genomic analyses.

In addition to these known mechanisms of persistence, colonization, and infection, additional currently un-identified mechanisms likely play a role in the pathogenesis of *A. baumannii* and related isolates. In this study, transcriptional regulators have been identified that are unique to specific nodes in the *Acb* complex and have unknown regulatory function (Table 1); several of these regulators share homology with regulators in other pathogens, which suggests a role of these genes in *A. baumannii* pathogenesis. Characterization of these regulators with a global and an un-biased approach, such as RNA-sequencing [90], may be required in order to better understand the regulatory networks of *A. baumannii*; these experiments are currently on-going.

Population level whole genome sequencing has provided a comprehensive tool for the analysis of *Acinetobacter* evolution. Furthermore, the established whole genome phylogeny has provided insight into important evolutionary relationships that cannot be fully determined with single gene or MLST analysis. The comparative method described in this study demonstrates how whole genome sequence analysis can be used to study the flow of genomic information between species in a genus, thereby allowing for an understanding of the evolution of an environmental microbe into a nosocomial pathogen. Guided by a whole-genome phylogeny, this flow of information can then be used to focus studies on the functional characterization of novel genomic features and determine their role in pathogenesis. This method represents a new paradigm in the identification and characterization of emerging human pathogens, such as *A. baumannii*.

## Supporting Information

Figure S1 **A phylogenetic tree inferred from an alignment of 16S rRNA gene sequences.** The tree was inferred with FastTree2 with 1000 bootstrap replicates; bootstrap support values are shown at nodes. Clades were collapsed in ARB [63].
(TIF)

Figure S2 **A comparison of phylogenetic trees inferred on either a whole genome alignment, or an alignment of concatenated multi-locus sequencing typing (MLST) (pubmlst.org/abaumannii) sequences informatically extracted from each genome analyzed in this study.** Trees were inferred with FastTree2 [52]. Clades were colored based on assignments made from the whole genome phylogeny.
(TIF)

Table S1 **Details of isolation, assembly, and accession of isolates sequenced in this study.**
(PDF)

Table S2 **Accession details of reference genomes analyzed in this study.**
(PDF)

Table S3 **Accession and annotation details for genes screened in this study.**
(PDF)

Table S4 **Raw blast score ratio (BSR) for each gene screened in this study.**
(PDF)

Table S5 **Homology of acquired genes in the Acinetobacter calcoaceticus-baumannii (Acb) complex to genes in other bacteria.**
(PDF)
