# Supplementary figures and images for: Evolution of a Pathogen: A Comparative Genomics Analysis Identifies a Genetic Pathway to Pathogenesis in Acinetobacter
Source: PLoS One. 2013 Jan 24;8(1):e54287. doi: 10.1371/journal.pone.0054287 (PMC3554770; doi:10.1371/journal.pone.0054287)

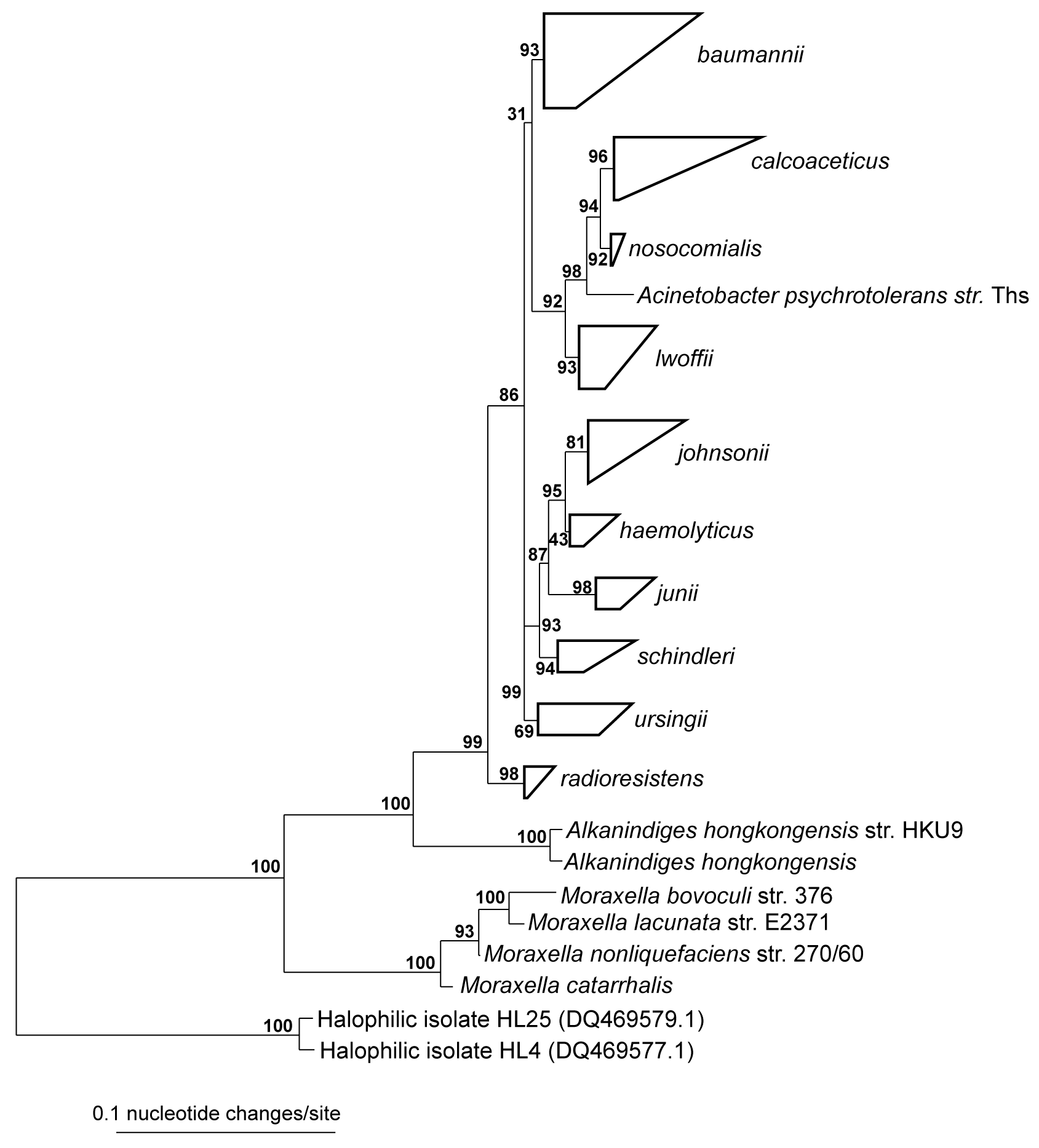

Supplement: Figure S1 — A phylogenetic tree inferred from an alignment of 16S rRNA gene sequences. The tree was inferred with FastTree2 with 1000 bootstrap replicates; bootstrap support values are shown at nodes. Clades were collapsed in ARB [63]. (TIF) [file pone.0054287.s001.tif]

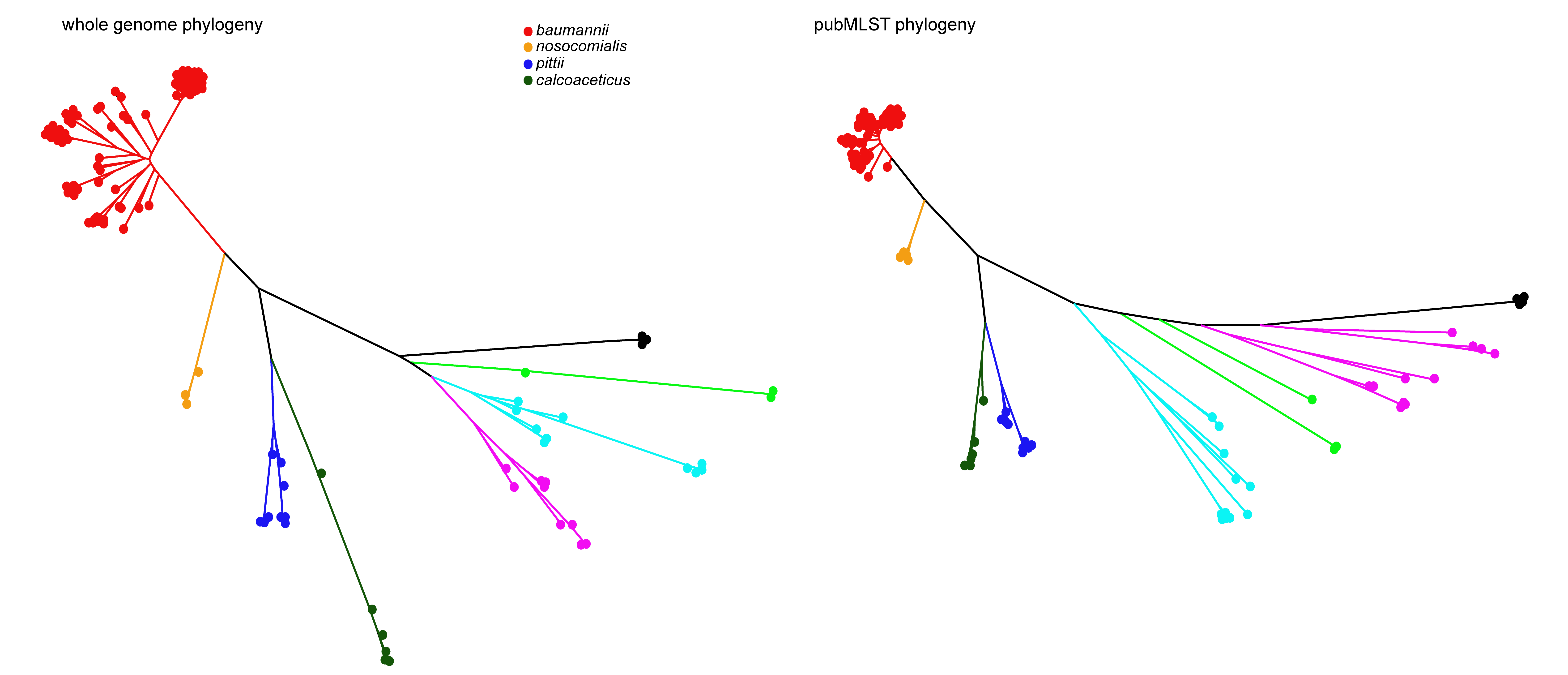

Supplement: Figure S2 — A comparison of phylogenetic trees inferred on either a whole genome alignment, or an alignment of concatenated multi-locus sequencing typing (MLST) (pubmlst.org/abaumannii) sequences informatically extracted from each genome analyzed in this study. Trees were inferred with FastTree2 [52]. Clades were colored based on assignments made from the whole genome phylogeny. (TIF) [file pone.0054287.s002.tif]
