# Supplementary material for: Evolution of a Pathogen: A Comparative Genomics Analysis Identifies a Genetic Pathway to Pathogenesis in Acinetobacter
Source: PLoS One. 2013 Jan 24;8(1):e54287. doi: 10.1371/journal.pone.0054287 (PMC3554770; doi:10.1371/journal.pone.0054287)
Supplement: Table S1 — Details of isolation, assembly, and accession of isolates sequenced in this study. (PDF) [file pone.0054287.s003.pdf]

Table S1. Details of isolation and assembly of isolates sequenced in this study

Genome (T)	#contigs	N50 (nts)	assembly size (nts)	isolation source	culture date	accession	Source*	External ID
A. baylyi TG19579	95	210650	3746897	soil	1961	AMIC00000000	ATCC	33304
A. calcoaceticus TG19585	34	370467	3810918	soil	1952	AMIW00000000	ATCC	17902
A. calcoaceticus TG19588	31	844197	3902769	unknown	unknown	AMIX00000000	ATCC	19638
A. calcoaceticus TG19593	29	369028	3911510	soil	1962	AMIY00000000	ATCC	14987
A. haemolyticus TG19599	261	36895	3386441	sputum	1962	AMJA00000000	ATCC	17906
A. haemolyticus TG19602	382	35130	3578946	ocular pus	unknown	AMJB00000000	ATCC	19002
A. haemolyticus TG21157	175	34302	3324307	cervix	1969	AMJC00000000	ATCC	27244
A. johnsonii TG19605	186	58817	3532488	duodenum	1962	AMJD00000000	ATCC	17909
A. johnsonii TG19625	111	65492	3599403	Venous catheter	1989	AMJE00000000	ATCC	700472
A. junii TG19608	136	51258	3257504	urine	1962	AMJF00000000	ATCC	17908
A. lwoffii TG19636	245	41518	3497784	urine	1962	AMJG00000000	ATCC	17910
A. nosocomialis TG19596	86	176764	3790318	unknown	unknown	AMIZ00000000	ATCC	BAA-347
A. nosocomialis TG21145	39	399595	4103207	unknown	1968	AMJH00000000	ATCC	17903
A. pittii TG6411	66	125920	3919746	sputum	2007	AMJI00000000	LSA	ACBA-36
A. radioresistens TG02010	38	168129	3063804	blood	2005	AMJJ00000000	LSA	ACBA-2
A. schindleri TG19614	159	65440	3197027	urine	1998	AMJK00000000	ATCC	BAA-618
A. sp. 528	293	35578	4051342	sputum	unknown	AMJL00000000	ASL	ID06-528
A. sp. TG19627	109	159110	4524664	urine	1962	AMJM00000000	ATCC	17988
A. sp. TG2027	66	242604	3927238	wound	2001	AMJN00000000	LSA	ACBA-27
A. baumannii 1536-8	194	44122	3844188	unknown	2006	AMHA00000000	ASL	ID06-1536
A. baumannii 1582-8	253	41921	4054837	sputum	2006	AMHB00000000	ASL	ID06-1582
A. baumannii 1583-8	322	29456	3889613	unknown	2006	AMHC00000000	ASL	ID06-1583
A. baumannii 1594-8	222	40465	3973075	sputum	2006	AMHD00000000	ASL	ID06-1594
A. baumannii 1595-8	205	44166	4070281	urine	2006	AMHE00000000	ASL	ID06-1595
A. baumannii 1649-8	140	54971	3786021	sputum	2006	AMHF00000000	ASL	ID06-1649
A. baumannii 1650-8	133	55744	3791740	hip	2006	AMHG00000000	ASL	ID06-1650
A. baumannii 1766-8	222	47166	4010538	body fluid	2006	AMJO00000000	ASL	ID06-1766
A. baumannii 2007-09-110-01-7	274	34327	3965851	hospital surface	2007	AMHH00000000	CDC	2007-09-110-01
A. baumannii 2007-16-25-01-7	81	135573	3961436	hospital surface	2007	AMHI00000000	CDC	2007-16-25-01
A. baumannii 2007-16-27-01	71	157349	3970108	hospital surface	2007	AMHJ00000000	CDC	2007-16-27-01
A. baumannii 2008-15-34-7	157	77102	3918675	sputum	2008	AMHK00000000	CDC	2008-15-34
A. baumannii 2008-15-45	140	83014	4100732	bronchoalveolar lavage	2008	AMHL00000000	CDC	2008-15-45
A. baumannii 2008-15-52	104	119999	3889383	wound	2008	AMHM00000000	CDC	2008-15-52
A. baumannii 2008-15-69	75	127651	4136907	unknown	2008	AMHN00000000	CDC	2008-15-69
A. baumannii 2008-15-70	234	55952	4101914	tracheal aspirate	2008	AMHO00000000	CDC	2008-15-70
A. baumannii 2008-15-71	90	140379	3886101	sputum	2008	AMHP00000000	CDC	2008-15-71
A. baumannii 2008-23-01-01-7	116	84761	4032165	hospital surface	2008	AMHQ00000000	CDC	2008-23-01-01
A. baumannii 2008-23-07-01-7	242	39941	3995020	hospital surface	2008	AMHR00000000	CDC	2008-23-07-01
A. baumannii 2009-04-01-7	86	103749	3839270	wound	2009	AMHS00000000	CDC	2009-04-01
A. baumannii 2009-04-02-7	163	55248	3972033	sputum	2009	AMHT00000000	CDC	2009-04-02
A. baumannii 515-8	222	42260	4077480	sputum	2006	AMHU00000000	ASL	ID06-515
A. baumannii 908-12	115	94610	3992622	hospital surface	2007	AMHV00000000	CDC	908-12
A. baumannii 908-13	175	75548	3973172	urine	2007	AMHW00000000	CDC	908-13
A. baumannii 908-14-7	291	31621	3850660	bronchoalveolar lavage	2007	AMHX00000000	CDC	908-14
A. baumannii 909-01-7	117	76009	3790690	blood	2007	AMHY00000000	CDC	909-01
A. baumannii 909-02-7	98	138985	3929638	sputum	2007	AMHZ00000000	CDC	909-02
A. baumannii 909-05	181	54820	4045600	sputum	2007	AMIA00000000	CDC	909-05
A. baumannii 909-14-7	584	12718	4006324	wound	2007	AMIB00000000	CDC	909-14
A. baumannii TG19582	366	20533	3797693	unknown	unknown	AMIV00000000	ATCC	BAA-346
A. baumannii TG19617	59	335015	3890586	unknown	unknown	AMID00000000	LSA	ACBA-22
A. baumannii TG2018	147	84900	3975196	sputum	2006	AMIE00000000	LSA	ACBA-11
A. baumannii TG2022	198	42438	3936090	blood	2006	AMIF00000000	LSA	ACBA-18
A. baumannii TG2023	175	49451	3944281	blood	2006	AMIG00000000	LSA	ACBA-20
A. baumannii TG2026	691	43885	4208784	wound	2006	AMIH00000000	LSA	ACBA-26
A. baumannii TG2028	129	57768	3770742	sputum	2001	AMII00000000	LSA	VEN-078
A. baumannii TG2030	151	87911	3764392	sputum	2001	AMIJ00000000	LSA	VEN-98
A. baumannii TG2031	75	140361	3782788	sputum	2001	AMIK00000000	LSA	VEN-104
A. baumannii TG2032	353	22057	3557150	sputum	2006	AMIL00000000	LSA	VEN-106
A. baumannii TG2631	95	107953	3881302	blood	2007	AMIM00000000	LSA	ACBA-28
A. baumannii TG27323	103	177990	3962171	unknown	2005	AMIN00000000	ASL	ID06-0170000521-001-A
A. baumannii TG27327	104	161007	4012960	wound	2005	AMIO00000000	ASL	ID06-0170000523-001-A
A. baumannii TG27331	148	140920	4062196	sputum	2005	AMIP00000000	ASL	ID06-0170000524-001-A
A. baumannii TG27335	137	143627	4061401	unknown	2005	AMIQ00000000	ASL	ID06-0170000525-001-A
A. baumannii TG27339	46	235222	3971513	sputum	2005	AMIR00000000	ASL	ID06-0170000526-001-A
A. baumannii TG27343	182	102490	4011165	wound	2005	AMIS00000000	ASL	ID06-0170000527-001-A
A. sp. TG27347	90	133924	4016748	sputum	2005	AMIT00000000	ASL	ID06-0170000528-001-A
A. baumannii TG5064	471	18081	3760971	blood	2007	AMIU00000000	LSA	ACBA-35

*CDC=Centers for Disease Control, ATCC=American Type Culture Collection, ASL=Arizona State Labs, LSA= Laboratory Sciences of Arizona
T = Type strain
