# Supplementary material for: Evolution of a Pathogen: A Comparative Genomics Analysis Identifies a Genetic Pathway to Pathogenesis in Acinetobacter
Source: PLoS One. 2013 Jan 24;8(1):e54287. doi: 10.1371/journal.pone.0054287 (PMC3554770; doi:10.1371/journal.pone.0054287)
Supplement: Table S2 — Accession details of reference genomes analyzed in this study. (PDF) [file pone.0054287.s004.pdf]

Table S2. Accession details of reference genomes analyzed in this study

Genome	Accession
A. baumannii AB0057	NC_011586.1
A. baumannii AB056	NZ_ADGZ00000000
A. baumannii AB058	NZ_ADHA00000000
A. baumannii AB059	NZ_ADHB00000000
A. baumannii 1656-2	CP001921.1
*A. baumanni*i AB210	AEOX00000000
A. baumannii AB307-0294	NC_011595.1
A. baumannii 3909	AEOZ00000000
A. baumannii 3990	AEOY00000000
A. baumannii 4190	AEPA00000000
A. baumannii 4857	AHAG00000000
A. baumannii 5075	AHAH00000000
A. baumannii 5256	AHAI00000000
A. baumannii 5711	AHAJ00000000
A. baumannii 6013113	NZ_ACYR00000000
A. baumannii 6013150	NZ_ACYQ00000000
A. baumannii 6014059	NZ_ACYS00000000
A. baumannii AB900	NZ_ABXK00000000
A. baumannii ACICU	NC_010611.1
A. baumannii MDR-TJ	AEOE00000000
A. baumannii TCDC-AB0715	CP002522.1
A. baumannii ATCC 19606	ACQB00000000
A. baumannii AYE	NC_010410.1
A. baumannii UMB001	AEPK00000000
A. baumannii UMB002	AEPL00000000
A. baumannii UMB003	AEPM00000000
A. baumannii A118	AEOW00000000
A. baumannii ABNIH1	AFSZ00000000
A. baumannii ABNIH2	AFTA00000000
A. baumannii ABNIH3	AFTB00000000
A. baumannii ABNIH4	AFTC00000000
A. baumannii ATCC 17978	CP000521.1
A. baumannii D1279779	AERZ00000000
A. baumannii MDR-ZJ06	CP001937.1
A. baumannii Naval-18	AFDA00000000
A. baumannii Naval-81	AFDB00000000
A. baumannii OIFC032	AFCZ00000000
A. baumannii W6976	AIEG00000000
A. baumannii W7282	AIEH00000000
A. baumannii WM99c	AERY00000000
A. baumannii SDF	NC_010400.1
A. bereziniae LMG 1003	AIEI00000000
A. calcoaceticus RUH2202	ACPK00000000
A. calcoaceticus DSM 30006	AIEC00000000
A. calcoaceticus PHEA-2	NC_016603.1
A. genomosp. 3 str. DSM 21653	AIEK00000000
A. genomosp. 3 str. DSM 9306	AIEF00000000
A. haemolyticus ATCC 19194	ADMT00000000
*A. johnson*ii SH046	ACPL00000000
A. junii SH205	ACPM00000000
A. lwoffii NCTC 5866	AIEL00000000
A. lwoffii SH145	ACPN00000000
*A. lwoffi*i WJ10621	NZ_AFQY00000000
A. genomosp. 13TU str. NCTC 8102	AIEJ00000000
A. oleivorans DR1	NC_014259.1
A. parvus DSM 16617	AIEB00000000
A. pittii D499	AGFH00000000
A. radioresistens DSM 6976	AIDZ00000000
A. radioresistens SK82	ACVR00000000
A. radioresistens SH164	ACPO00000000
A. sp. ADP1	NC_005966.1
A. sp. ATCC 27244	ABYN00000000
A. sp. NBRC 100985	BAEB00000000
A. sp. NCTC 10304	AIEE00000000
A. sp. NCTC 7422	AIED00000000
A. sp. P8-3-8	AFIE00000000
A. sp. RUH2624	ACQF00000000
A. sp. SH024	ADCH00000000
A. ursingii DSM 16037	AIEA00000000
