# Supplementary material for: Evolution of a Pathogen: A Comparative Genomics Analysis Identifies a Genetic Pathway to Pathogenesis in Acinetobacter
Source: PLoS One. 2013 Jan 24;8(1):e54287. doi: 10.1371/journal.pone.0054287 (PMC3554770; doi:10.1371/journal.pone.0054287)
Supplement: Table S3 — Accession and annotation details for genes screened in this study. (PDF) [file pone.0054287.s005.pdf]

Table S3. Accession and annotation details for genes screened in this study

Locus tag	Annotation
ABAYE1085	acinetobactin utilization protein BauF
ABAYE1086	acinetobactin biosynthesis protein BasA
ABAYE1087	non-ribosomal peptide synthase
ABAYE1089	ferric acinetobactin transport system permease
ABAYE1090	ferric acinetobactin transport system permease
ABAYE1091	ferric acinetobactin transport system ATP-binding protein
ABAYE1092	ferric acinetobactin binding protein (BauB)
ABAYE1093	ferric acinetobactin receptor (bauA)
ABAYE1094	acinetobactin siderophore biosynthesis protein BasC
ABAYE1095	non-ribosomal peptide synthase (BasD)
ABAYE1096	enterobactin synthase subunit E (BasE)
ABAYE1102	thioesterase
ABAYE1103	phosphopantetheinyl transferase component of acinetobactin biosynthesis (basI)
ABAYE1104	isochorismate synthetase
ABAYE2001	ferric siderophore receptor protein
ABAYE2002	demethylmenaquinone methyltransferase
ABAYE2003	hypothetical protein
ABAYE2004	siderophore biosynthesis protein
ABAYE2005	hypothetical protein
ABAYE2006	MFS superfamily multidrug resistance protein
ABAYE2007	lysine/ornithine N-monooxygenase
ABAYE2008	siderophore biosynthesis protein
ABAYE1644	ferrisiderophore receptor protein, TonB dependent
BAM0936	putative acinetoferrin biosynthesis protein
BAM0937	putative acinetoferrin biosynthesis protein
BAM0938	putative acinetoferrin biosynthesis protein
BAM0939	putative acinetoferrin biosynthesis protein
BAM0940	hypothetical protein
BAM0941	putative ferric acinetoferrin transporter
BAM0942	putative ferric acinetoferrin receptor
BAM0943	putative acinetoferrin exporter
pABIRp15	OXA-58 oxacillinase
ABX45066	beta-lactamase OXA-51-like protein
ACC97561	blaOXA-23
AB57_0550	transposase
AB57_2080	multidrug efflux protein AdeC
ABK1_3039	multidrug efflux protein AdeJ
AB57_3158	multidrug efflux protein AdeK
AB57_3156	multidrug efflux protein AdeI
ABAYE1173	multidrug ABC transporter
29_161	RND family drug transporter
29_170	RND family drug transporter
ABAYE1176	LysR family transcriptional regulator
32_436	multidrug efflux protein AdeB
32_431	AdeA membrane fusion protein
ABX83929	AdeR
ABX83930	AdeS
IPREF0010_0184	multidrug efflux pump AbeM
