# Supplementary material for: Evolution of a Pathogen: A Comparative Genomics Analysis Identifies a Genetic Pathway to Pathogenesis in Acinetobacter
Source: PLoS One. 2013 Jan 24;8(1):e54287. doi: 10.1371/journal.pone.0054287 (PMC3554770; doi:10.1371/journal.pone.0054287)
Supplement: Table S5 — Homology of acquired genes in the Acinetobacter calcoaceticus-baumannii (Acb) complex to genes in other bacteria. (PDF) [file pone.0054287.s007.pdf]

Table S5. Homology of acuired genes in the *Acinetobacter calcoaceticus-baumannii* (*Acb*) complex to genes in other bacteria

feature	locus_tag	Closest BLAST hit	ID (%)	coverage (%)	Closest BLAST hit type
1	ABAYE2283	*Azotobacter vinelandii* DJ	49	92	soil microbe
2	ABAYE0080	*Rhodanobacter* sp. 2APBS1	67	92	soil microbe
3	ABAYE3752	*Spirochaeta smaragdinae*	25	92	soil microbe
4	ABAYE3753	*Verminephrobacter eiseniae*	43	86	earthworm endosymbiont
5	ABAYE3761	*Methylobacter tundripaludum*	59	97	soil microbe
6	ABAYE2829	*Zymomonas mobilis*	54	93	soil microbe
7	ABAYE1524	*Serratia* sp. M24T3	44	89	nematode symbiont
8	ABAYE1477	*Yersinia frederiksenii*	53	89	pathogen
9	ABAYE2143	*Naumovozyma dairenensis*	30	35	fungus
10	ABAYE0743	*Microvirga* sp. WSM3557	34	98	root nodulating bacterium
11	ABAYE1358	*Pseudomonas syringae*	49	92	pathogen
12	ABAYE1778	*Gluconobacter morbifer*	46	97	pathogen
13	ABAYE0223	*Chryseobacterium gleum*	59	94	pathogen
14	ACICU_01049	*Callithrix jacchus*	31	76	marmoset
15	ABAYE2708	*Marinobacter adhaerens* HP15	47	49	diatom aggregate
16	HMPREF0022_02462	*Schizophyllum commune*	37	58	fungus
17	ACICU_02424	*Methylotenera mobilis*	48	97	sediment microbe
18	ABAYE1316	*Pseudomonas stutzeri*	39	96	pathogen
19	ABAYE2003	*Bacillus aerophilus*	27	83	environmental microbe
20	AbauAB05_010100017737	*Clostridium difficile*	38	80	pathogen
21	AbauAB059_010100020508	No hit	N/A	N/A	N/A
22	HMPREF0022_00070	*Commensalibacter intestini*	44	59	gut
23	ABZJ_00026	*Arthrobotrys oligospora*	42	51	fungus
24	AbauAB0_010100001652	*Plasmodium vivax*	26	74	pathogen
25	ABAYE2456	Azospirillum brasilense	43	84	soil microbe
26	HMPREF0021_00524	No hit	N/A	N/A	N/A
27	ABAYE0983	*Serpula lacrymans*	26	67	fungus
28	ABAYE1931	*Burkholderia gladioli*	56	100	pathogen
29	HMPREF0012_03541	*Dichelobacter nodosus*	75	100	pathogen
30	HMPREF0012_00124	*Mesorhizobium alhagi*	53	99	soil microbe
31	HMPREF0012_00560	*Methylophaga aminisulfidivorans*	57	92	marine microbe
32	HMPREF0012_00562	Rhodoferax ferrireducens	53	90	environmental microbe
33	HMPREF0023_2071	Gordonia araii	55	99	pathogen
34	HMPREF0013_03409	Klebsiella oxytoca	64	100	pathogen
35	HMPREF0013_03179	Serratia plymuthica	77	97	opportunistic pathogen
36	HMPREF0013_03184	Psychrobacter sp. 1501	52	98	pathogen
37	HMPREF0012_01063	Myroides odoratus	74	97	pathogen
38	HMPREF0026_02170	Pseudomonas syringae	68	89	pathogen
39	HMPREF0026_00688	Pseudomonas aeruginosa	50	89	pathogen
40	HMP0015_2881	Brenneria sp. EniD312	28	77	pathogen
41	HMPREF0026_01273	Psychrobacter arcticus	54	92	soil microbe
42	HMPREF0023_2956	Alcanivorax borkumensis	52	98	marine microbe
