# Supplementary material for: Evolution of a Pathogen: A Comparative Genomics Analysis Identifies a Genetic Pathway to Pathogenesis in Acinetobacter
Source: PLoS One. 2013 Jan 24;8(1):e54287. doi: 10.1371/journal.pone.0054287 (PMC3554770; doi:10.1371/journal.pone.0054287)
Supplement: Table S4 — Raw blast score ratio (BSR) for each gene screened in this study. (PDF) [file pone.0054287.s006.pdf]

Table S4. Raw blast score ratio (BSR) for each gene screened in this study

genome	ABAYE2283	ABAYE0080	ABAYE3752	ABAYE3753	ABAYE3761	ABAYE2829	ABAYE1524	ABAYE1477	ABAYE2143	ABAYE0743	[illegible]
A_baylyi_TG19579	0.0000	0.0232	0.0305	0.0156	0.0789	0.0372	0.1002	0.0715	0.0982	0.0812	[illegible]
A_bereziniae_LMG1003	0.0000	0.0244	0.0388	0.0145	0.0855	0.0000	0.0000	0.0000	0.0982	0.0888	[illegible]
A_calcoaceticus_DSM_30006	0.8650	0.9604	0.9175	0.9538	0.9653	0.9652	0.8880	0.8848	0.8650	0.9604	[illegible]
A_calcoaceticus_TG19585	0.8650	0.9620	0.9129	0.9533	0.9740	0.9626	0.8798	0.8848	0.8650	0.9604	[illegible]
A_calcoaceticus_TG19588	0.8650	0.9604	0.9175	0.9538	0.9653	0.9652	0.8880	0.8848	0.8650	0.9604	[illegible]
A_calcoaceticus_TG19593	0.8624	0.9684	0.9187	0.9529	0.9740	0.9601	0.8728	0.8848	0.8650	0.0878	[illegible]
A_haemolyticus_TG19599	0.0000	0.0229	0.0317	0.0143	0.0847	0.0000	0.0000	0.0644	0.0942	0.0878	[illegible]
A_haemolyticus_TG19602	0.0000	0.0229	0.0348	0.0143	0.0711	0.0397	0.0314	0.0686	0.0942	0.0914	[illegible]
A_haemolyticus_TG21157	0.0339	0.0000	0.0317	0.0143	0.0835	0.0347	0.0337	0.0644	0.0996	0.0825	[illegible]
A_johnsonii_SH046	0.0358	0.0222	0.0300	0.0140	0.0734	0.0000	0.0342	0.0000	0.0927	0.0901	[illegible]
A_johnsonii_TG19605	0.0000	0.0222	0.0000	0.0194	0.0000	0.0357	0.0342	0.0000	0.0927	0.0888	[illegible]
A_johnsonii_TG19625	0.0000	0.0222	0.0000	0.0194	0.0000	0.0357	0.0342	0.0000	0.0927	0.0888	[illegible]
A_junii_SH205	0.0387	0.0229	0.0326	0.0121	0.0913	0.0000	0.0328	0.0704	0.0942	0.0914	[illegible]
A_junii_TG19608	0.0339	0.0232	0.0308	0.0126	0.0867	0.0347	0.0319	0.0704	0.0942	0.0865	[illegible]
A_lwoffii_NCTC_5866	0.0343	0.0000	0.0336	0.0142	0.0000	0.0367	0.0342	0.0665	0.0971	0.0901	[illegible]
A_lwoffii_SH145	0.0353	0.0000	0.0336	0.0142	0.0945	0.0367	0.0342	0.0696	0.0971	0.0914	[illegible]
A_lwoffii_TG19636	0.0353	0.0222	0.0300	0.0142	0.0867	0.0362	0.0337	0.0686	0.0971	0.0914	[illegible]
A_lwoffii_WJ10621	0.0000	0.0237	0.0300	0.0180	0.0000	0.0000	0.0342	0.0704	0.0927	0.0878	[illegible]
A_nosocomialis_TG19596	0.9070	0.9707	0.0339	0.0157	0.9624	0.9858	0.8390	0.9503	0.9599	0.9670	[illegible]
[illegible]	[illegible]	[illegible]	[illegible]	[illegible]	[illegible]	[illegible]	[illegible]	[illegible]	[illegible]	[illegible]	[illegible]
